# Production of Structured Phosphatidylcholine with High Content of DHA/EPA by Immobilized Phospholipase A_1_-Catalyzed Transesterification

**DOI:** 10.3390/ijms150915244

**Published:** 2014-08-28

**Authors:** Xiang Li, Jia-Feng Chen, Bo Yang, Dao-Ming Li, Yong-Hua Wang, Wei-Fei Wang

**Affiliations:** 1 College of Light Industry and Food Sciences, South China University of Technology, Guangzhou 510641, China; E-Mails: lxgryx@126.com (X.L.); chenjiafeng@163.com (J.-F.C.); logangryx@163.com (D.-M.L.); 2 School of Bioscience and Bioengineering, South China University of Technology, Guangzhou 510006, China; E-Mail: yangbo@scut.edu.cn

**Keywords:** immobilized phospholipsase A_1_, structured phosphatidylcholine, docosahexaenoic, eicosapentaenoic

## Abstract

This paper presents the synthesis of structured phosphatidylcholine (PC) enriched with docosahexaenoic acid (DHA) and eicosapentaenoic acid (EPA) by transesterification of DHA/EPA-rich ethyl esters with PC using immobilized phospholipsase A_1_ (PLA_1_) in solvent-free medium. Firstly, liquid PLA_1_ was immobilized on resin D380, and it was found that a pH of 5 and a support/PLA_1_ ratio (*w*/*v*) of 1:3 were the best conditions for the adsorption. Secondly, the immobilized PLA_1_ was used to catalyze transesterification of PC and DHA/EPA-rich ethyl esters. The maximal incorporation of DHA and EPA achieved was 30.7% for 24 h of reaction at 55 °C using a substrate mass ratio (PC/ethyl esters) of 1:6, an immobilized PLA_1_ loading of 15% and water dosage of 1.25%. Then the reaction mixture was analyzed by ^31^P nuclear magnetic resonance (NMR). The composition of reaction product included 16.5% PC, 26.3% 2-diacyl-*sn*-glycero-3-lysophosphatidylcholine (1-LPC), 31.4% 1-diacyl-*sn*-glycero-3-lysophosphatidylcholine (2-LPC), and 25.8% *sn*-glycerol-3-phosphatidylcholine (GPC).

## 1. Introduction

Structured phospholipids (SPL) attract attention because of their special function in food, cosmetics and pharmaceutical industries [1]. With the development of society, more and more people start paying attention to health. Therefore, markets demand more SPL with novel functional and nutraceutical properties. The constituents and concentrations of incorporated fatty acids in the SPL’s scaffold can influence their physiological, physiochemical and biochemical characteristics. Novel SPL could be created by incorporating functional fatty acids (e.g., docosahexaenoic acid (DHA), eicosapentaenoic acid (EPA)) into natural PL. Docosahexaenoic (DHA; C22:6) and eicosapentaenoic (EPA; C20:5), which are functional n-3 polyunsaturated fatty acids (PUFA), have multiple biochemical and pharmacological effects on human health and treatment/prevention of some diseases [2,3,4]. It has been demonstrated that n-3 PUFA could be easily absorbed and have higher bioavailability in the body as PL than as the corresponding triglycerides or ethyl esters [5]. Moreover, Cansell* et al.* [6] have reported that DHA is less subjected to peroxidation when linked to phospholipids (PL) than to triglycerides or ethyl esters. Thus, more research on DHA/EPA-rich SPL will benefit its production.

Enzymatic processes to manufacture SPL–DHA/EPA are effective because of mild reaction conditions, specific substrate and positional selectivity, high catalytic efficiency, less side products and toxic material. In the process of preparing DHA/EPA-rich SPL, reactions such as acidolysis and transesterification were used to modify the natural PL, and both phospholipases and lipases could be used as biocatalysts. Several studies have been published concerning the modification of PL by enzymatic method (Table 1). Phospholipase A_1_ (PLA_1_) and phospholipase A_2_ (PLA_2_) showed better catalytic efficiency (incorporation ≥ 20%), whereas lipases such as Lipozyme RM IM (*Rhizomucor miehei* lipase immobilized on ion-exchange resin) and Lipozyme TL IM (*Thermomyces lanuginosus* lipase immobilized on silica gel) showed 18.9% incorporation in acidolysis [7] and 12.3% incorporation in transesterification [8]. Compared with the acidolysis reaction (free fatty acids and PL), the transesterification reaction (e.g., PL or lyso-PL and fish oil) could provide good solubility of reaction substrates and benefit reaction proceeding. Thus, for the production of SPL with high content of DHA and EPA, it is a good reaction system that PL was modified by transesterification of natural PL and DHA/EPA-rich ethyl esters without solvent as solubilizer. However, few studies on transesterification to produce DHA/EPA-rich SPL have been reported, thus more studies on it will be indispensible to develop better catalytic processes.

The aim of the present work was to produce structured phosphotidylcholine (PC) with high content of DHA and EPA. Firstly, the liquid PLA_1_ was immobilized by physical absorption on resin, and then the immobilized one was used to catalyze the transesterification of PC and DHA/EPA-rich ethyl esters in a solvent-free system. The reaction parameters, such as enzyme loading, substrate mass ratio, reaction temperature and pH, were investigated to evaluate the reaction efficiency, and the reusability of the immobilized PLA_1_ was also evaluated. Finally, the composition of structured DHA/EPA-rich PC was analyzed by ^31^P NMR.

**Table 1:** Synthesis of n-3 PUFA-rich phospholipids by enzyme-catalyzed reactions.

Process	Incorporation (%)	Reaction Substrate	Enzyme Load	System	Reference
Transesterification	12.3	Ethyl esters ^a^/PL	10% Lipozyme RM IM	Hexane	[8]
Acidolysis	43	FFA ^b^/PC	15% Immobilized PLA_1_	Solvent-free	[9]
Acidolysis	35	FFA ^c^/PC	10% Immobilized PLA_1_	Solvent-free	[10]
Acidolysis	28	FFA ^b^/PC	10% Liquid PLA_1_	Solvent-free	[11]
Acidolysis	20	FFA ^d^/PC	30% Immobilized PLA_2_	Solvent-free	[12]
Acidolysis	18.9	FFA ^e^/PL	20% Lipozyme TL IM	Solvent-free	[7]

^a^ 52% eicosapentaenoic acid (EPA) and 20% docosahexaenoic acid (DHA); ^b^ 12.2% EPA, 10.1% docosapentaenoic acid (DPA) and 60.7% DHA; ^c^ 78.4% EPA + DPA + DHA; ^d^ purity > 99% DHA; ^e^ 35% EPA and 25% DHA. Abbreviations: PUFA, polyunsaturated fatty acids; Lipozyme RM IM, *Rhizomucor miehei* lipase immobilized on ion-exchange resin; PLA, Phospholipase A; Lipozyme TL IM, *Thermomyces lanuginosus* lipase immobilized on silica gel.

## 2. Results and Discussion

### 2.1. Immobilization of Phospholipase A_1_ (PLA_1_)

Immobilized enzyme benefits transesterification reactions because of its easy recycle and high economic and reaction efficiency. In this section, the immobilization of PLA_1_ was performed, and resin D380 was selected to be the support for the enzyme. D380 is a macroporous and weakly basic anion exchange resin and it could form ionic bonds between functional group (–NH_2_) and the amino acid residues in protein molecules, which is helpful to show higher enzyme activity [13]. Factors such as the ratio of support/PLA_1_ and pH were selected to investigate their effects on the protein load and the activity of immobilized PLA_1_.

#### 2.1.1. Effect of Support/PLA_1_ Ratio

**Figure 1:**
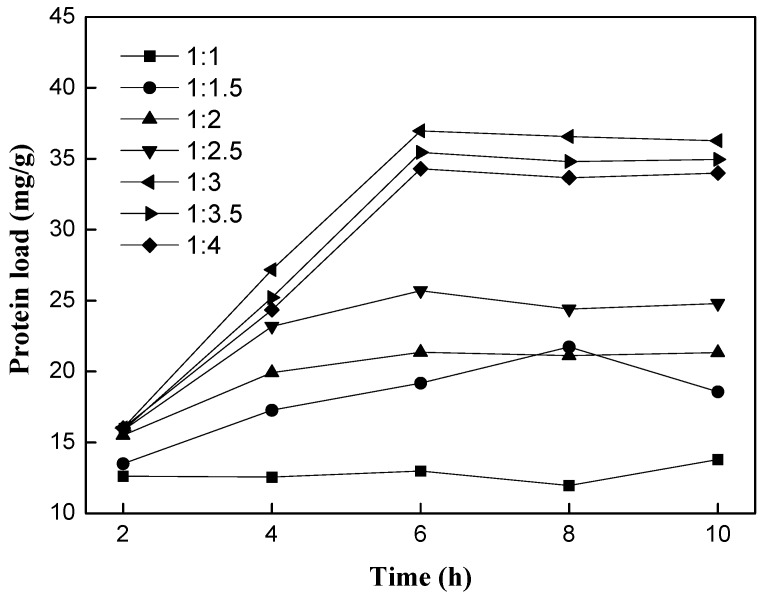
Effect of support/phospholipase A_1_ (PLA_1_) ratio (*w*/*v*) on protein load. Reaction conditions: resin D380, 2 g; temperature 30 °C; pH 7.0.

**Figure 2:**
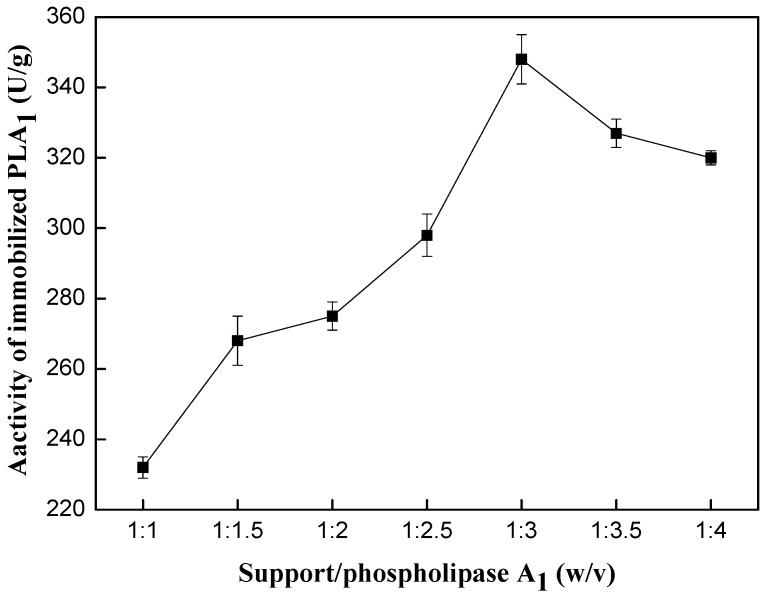
Effect of support/phospholipase A_1_ ratio (*w*/*v*) on activity of immobilized phospholipase A_1_. Reaction conditions: resin D380, 2 g; temperature, 30 °C; pH 7.0; absorption time, 6 h; substrate, soy lecithin (PC > 40%).

The protein load and activity of immobilized PLA_1_ were evaluated when support/PLA_1_ ratio (*w*/*v*) was changed. The support/PLA_1_ ratio (*w*/*v*) was set as 1:1, 1:1.5, 1:2, 1:2.5, 1:3, 1:3.5, 1:4, respectively. Support/PLA_1_ ratio (*w*/*v*) means the ratio of the weight (g) of support to the volume (mL) of a solution of PLA_1_ (containing 1.5% of protein). It could be seen from Figure 1 that the protein load increased with the ratio of support to PLA_1_ increasing from 1:1 to 1:3, and it achieved a maximum of 36.96 mg/g when the ratio of support to PLA_1_ was 1:3 with 6 h of incubation, and then decreased with the ratio rising (Figure 1). Furthermore, the activity of immobilized PLA_1_ showed good consistency with the effect of support/PLA_1_ ratio on protein load (Figure 2). The maximal phospholipase activity was 348.42 U/g at support to PLA_1_ ratio of 1:3. When the support/PLA_1_ ratio was higher than 1:3, the enzyme activity decreased. This can be explained by the multilayer adsorption at high enzyme load which can possibly block or inhibit access to enzyme active sites.

#### 2.1.2. Effect of pH

**Figure 3:**
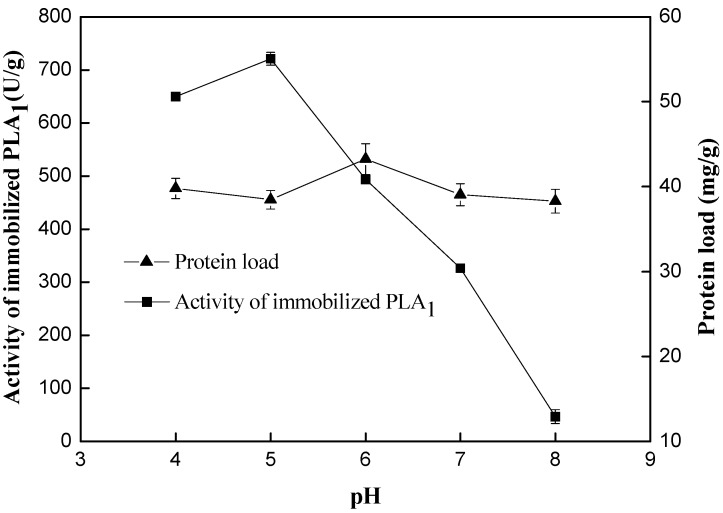
Effect of pH on activity and protein load of immobilized phospholipase A_1_. Reaction conditions: undiluted free phospholipase A_1_, 6 mL; phosphate buffer, 6 mL; resin D380, 2 g; temperature, 30 °C; absorption time, 6 h; substrate, soy lecithin (PC > 40%).

Effect of pH (4.0–8.0) on the activity of immobilized PLA_1_ was evaluated and the results are shown in Figure 3. pH showed less effect on the protein load, but had significant influence on phospholipase activity. It could be seen that the activity occurred mainly in the acidic region with a maximum of 720.95 U/g at pH 5.0. Activity of immobilized PLA_1_ decreased sharply when pH was over 5, suggesting that the PLA_1_ would be denatured in an alkaline environment. On the basis of this result and our desire to avoid potential denaturation of the enzyme with the concomitant loss of enzyme activity, we selected pH 5.0 for immobilization. The optimal pH is different from that reported by Garcia *et al.* [10]. However, Garcia *et al.* have reported that the maximum adsorption of the PLA_1_ was achieved under neutral conditions (pH 7). The differing results of optimal pH observed is likely due to different carriers used. In conclusion, the optimal immobilization conditions are: support/PLA_1_ ratio of 1:3 and pH of 5 at 30 °C for 6 h. Therefore, the immobilized PLA_1_ was employed in subsequent experiments.

#### 2.1.3. Catalyzing the Transesterification of PC and Docosahexaenoic Acid (DHA)/Eicosapentaenoic Acid (EPA)-Rich Ethyl Esters by Free PLA_1_ and Immobilized PLA_1_

**Figure 4:**
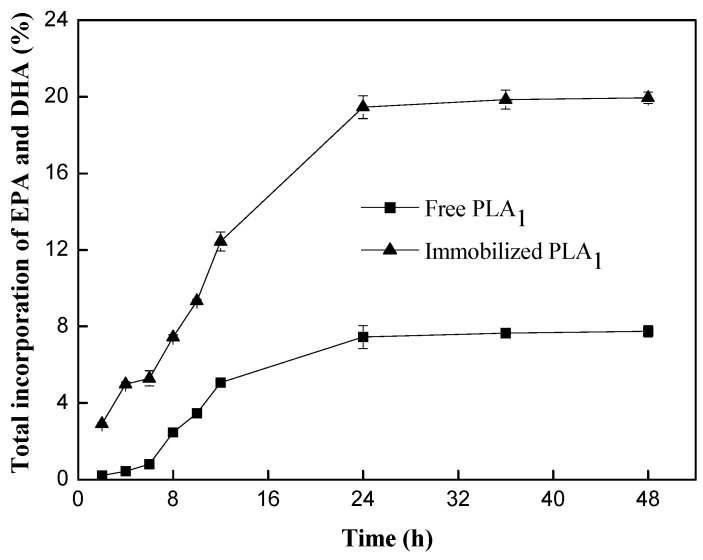
Effect of free PLA_1_ and immobilized PLA_1_ on the transesterification reaction. Reaction conditions: enzyme loading, 10% (*w*/*w*); substrate mass ratio (PC/ethyl esters), 1:4; temperature, 50 °C; water dosage, 1% (*w*/*w*).

The reaction was conducted at 50 °C with the enzyme loading of 10% (free PLA_1_ activity: 3000 U; immobilized PLA_1_ activity: 360.48 U), substrate mass ratio of 1:4 (PC to DHA/EPA-rich ethyl esters). The results showed that immobilized PLA_1_ exhibited higher catalytic capacity than free PLA_1_ did. A 19.5% of incorporation was observed for immobilized PLA_1_ at 24 h of reaction, whereas only 7.4% for free PLA_1_ (Figure 4). This can be explained that the free enzyme supplied by Novozymes A/S (Bagsværd, Denmark) was as an aqueous solution of PLA_1_ containing 56% water. A large amount of water presented in the system greatly accelerated the hydrolysis of PC during the transesterification of PC with ethyl esters, yielding more *sn*-glycerol-3-phosphatidylcholine (GPC). However, a small amount of water was added in the transesterification reaction catalyzed by the immobilized enzyme. Thus, the hydrolysis of PC would be less significant. Accordingly, the catalytic efficiency of the immobilized enzyme was greatly improved compared to that of the free one; similar results were observed by Kim *et al.* [9,10], where 43% incorporation was observed in the acidolysis reaction catalyzed by immobilized PLA_1_, whereas only 28% incorporation was observed in the acidolysis reaction catalyzed by free PLA_1_. Based on these results, the immobilized PLA_1_ was employed in subsequent experiments.

### 2.2. Transesterification of PC with DHA/EPA-Rich Ethyl Esters by Immobilized PLA_1_

**Scheme 1:**
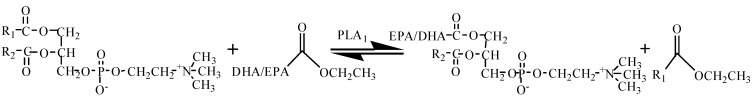
Transesterification reaction of PC with DHA/EPA-rich ethyl esters.

The immobilized PLA_1_ was prepared under the above optimized conditions and lyophilized. Its activity and moisture content were determined to be 720.95 U/g and 1.96%, respectively. Then the immobilized PLA_1_ was employed to catalyze the transesterification reaction of PC with DHA/EPA-rich ethyl esters (simplified reaction equation shown in Scheme 1), and reaction conditions, such as enzyme load, substrates mass ratio, reaction temperature and water dosage, were investigated.

#### 2.2.1. Effect of Enzyme Loading

**Figure 5:**
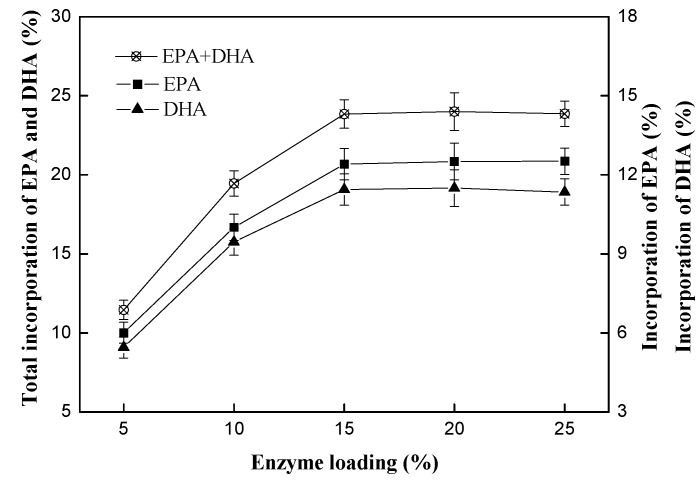
Effect of immobilized phospholipase A_1_ loading on the transesterification reaction. Reaction conditions: substrate mass ratio (PC: ethyl esters), 1:4; temperature, 50 °C; water dosage, 1% (*w*/*w*); reaction time, 24 h.

Effect of immobilized PLA_1_ loading on the transesterification reaction was evaluated. Enzyme loading is the percentage of the immobilized enzyme weight (containing enzyme itself and the resin D380) to the total weight of reaction substrates (PC and ethyl esters). The reaction was performed at 50 °C for 24 h with 1:4 of substrate mass ratio (PC to DHA/EPA-rich ethyl esters) and 1% of water dosage. The enzyme loading was selected as 5%, 10%, 15%, 20%, 25% (*w*/*w*, with respect to total reaction mixture), respectively. The results are shown in Figure 5. The incorporation of DHA and EPA into PC enhanced with the immobilized PLA_1_ load increasing. When >10% immobilized PLA_1_ was employed, the incorporation of DHA and EPA reached more than 19% after 24 h, and were 19.5% and 23.8% respectively with the enzyme loading at 10% and 15%. The optimal enzyme loading was 15%, and the incorporation of EPA and DHA were 12.4% and 11.4%, respectively.

#### 2.2.2. Effect of Mass Ratio of PC to DHA/EPA-Rich Ethyl Esters

**Figure 6:**
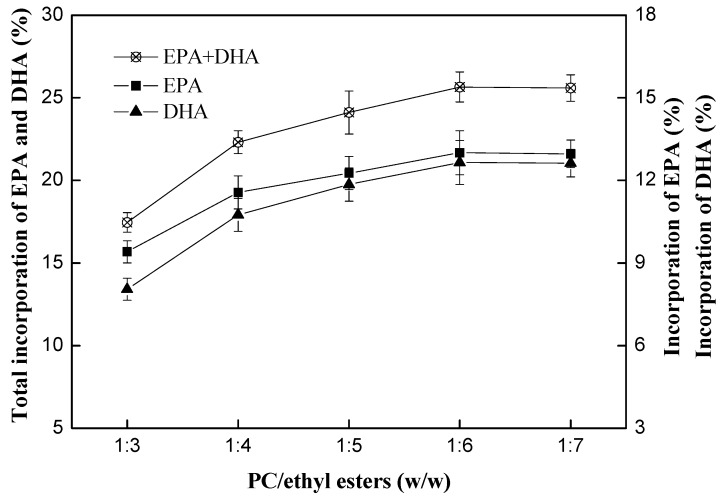
Effect of substrate mass ratio (PC/ethyl esters) on the transesterification reaction. Reaction conditions: immobilized phospholipase A_1_ loading, 15% (*w*/*w*); temperature, 50 °C; water dosage, 1% (*w*/*w*); reaction time, 24 h.

Effect of mass ratio of PC to DHA/EPA-rich ethyl esters on the transesterification reaction was investigated. The reaction was conducted at 50 °C with the enzyme loading of 15%. And the mass ratio of PC to DHA/EPA-rich ethyl esters was set as 1:3, 1:4, 1:5, 1:6 and 1:7, respectively. The results are shown in Figure 6. When the mass ratio of PC to DHA/EPA-rich ethyl esters varied from 1:3 to 1:6, the incorporation of DHA and EPA increased and it achieved a maximum of 25.6% at 1:6, whereas less increment was observed at 1:7. The incorporation of EPA and DHA respectively reached the maximum of 13.0% and 12.6% when substrate mass ratio was 1:6. Excess DHA/EPA-rich ethyl esters would benefit the incorporation of DHA/EPA into PC. This could be explained that excess DHA/EPA-rich ethyl esters might increase the concentration of DHA/EPA, the solubility of PC and decrease mass transfer. Therefore, the mass ratio was fixed at 1:6 in the latter reaction system.

#### 2.2.3. Effect of Temperature

Effect of temperature on the transesterification was explored. The temperature was set as 40, 45, 50, 55 and 60 °C, respectively. The enzyme loading was fixed at 15%, and mass ratio of PC to DHA/EPA-rich ethyl esters was kept at 1:6. The results are shown in Table 2. Compared with the initial ratio of EPA to DHA (0.84) in the DHA/EPA-rich ethyl esters, the ratio of EPA to DHA changed to be 1.2, 1.11, 1.04, 1.01, 0.98 after 24 h of reaction at 40, 45, 50, 55, 60 °C respectively. It was indicated that immobilized PLA_1_ showed slightly more preference to EPA than DHA whereas the tendency decreased with the reaction temperature rising. The incorporation of DHA/EPA into PC increased obviously with temperature rising and reached a maximum of 27.8% at 55 °C. When the temperature was over 55 °C, the incorporation was slightly enhanced, but not significantly (*p* < 0.05). Meanwhile, it is well known that the activity and stability of the immobilized enzyme would decrease faster after several reuse cycles at higher temperature. Therefore, the optimal temperature was fixed at 55 °C for the sake of the reusability of the enzyme, and the corresponding incorporation of EPA and DHA were 14.0% and 13.8%, respectively.

**Table 2:** Fatty acid composition of original DHA/EPA-rich ethyl esters and structured PC in transesterification reactions with different temperature.

Fatty Acid	DHA/EPA-Rich Ethyl Esters	Structured PC ^f^				
		40 °C	45 °C	50 °C	55 °C	60 °C
EPA (%)	38.1 ± 0.24	7.3 ± 0.28	10.1 ± 0.36	13.1 ± 0.39	14.0 ± 0.48	13.9 ± 0.58
DHA (%)	45.5 ± 0.31	6.1 ± 0.17	9.1 ± 0.29	12.6 ± 0.28	13.8 ± 0.39	14.2 ± 0.49
EPA + DHA (%)	83.6 ± 0.38	13.4 ± 0.42	19.2 ± 0.64	25.7 ± 0.58	27.8 ± 0.65	28.1 ± 0.71
Ratio of EPA to DHA	0.84 ± 0.01	1.20 ± 0.03	1.11 ± 0.03	1.04 ± 0.03	1.01 ± 0.04	0.98 ± 0.04

^f^ Reaction conditions: enzyme loading, 15% (*w*/*w*); substrate mass ratio (PC/ethyl esters), 1:6; water dosage (*w*/*w*), 1%; reaction time, 24 h.

#### 2.2.4. Effect of Water Dosage

Water content plays a crucial role in microaqueous phase reaction. A minimal amount of water must be present if the enzyme is to be present in the configuration that is quasi optimum from the standpoint of catalytic activity and selectivity [14]. Hence, effect of water dosage on the incorporation of DHA/EPA was investigated. The water dosage was set as 0.5%, 0.75%, 1%, 1.25% and 1.5%, respectively. The results are shown in Table 3. It was observed that the ratio of EPA to DHA decreased from 1.18 to 0.98 with the water dosage increased from 0.5% to 1.5%, suggesting that water dosage would affect the DHA/EPA incorporation in the transesterification. Moreover, a marked increase of the incorporation of DHA and EPA was observed as the water dosage increased from 0.25% to 1.25%, and the maximal content of DHA and EPA was achieved at 1.25% of water dosage, in which DHA and EPA content was 15.4% and 15.3%, respectively. When the water dosage was more than 1.25%, the incorporation of DHA and EPA decreased to be 27.5%, and this is mainly because excess water led to undesired hydrolysis reactions.

**Table 3:** Fatty acid composition of original DHA/EPA-rich ethyl esters and structured PC at different water dosage in transesterification reaction.

Samples	Fatty Acids Profiles (%)			Ratio of EPA to DHA
	EPA	DHA	DHA + EPA	
DHA/EPA-rich ethyl esters	38.1 ± 0.24	45.5 ± 0.31	83.6 ± 0.38	0.84 ± 0.01
Structured PC ^g^–0.5% ^h^	10.7 ± 0.29	9.1 ± 0.22	19.8 ± 0.45	1.18 ± 0.02
Structured PC–0.75%	12.7 ± 0.37	12.5 ± 0.32	25.2 ± 0.62	1.02 ± 0.01
Structured PC–1.0%	14.0 ± 0.28	13.9 ± 0.47	27.9 ± 0.51	1.01 ± 0.01
Structured PC–1.25%	15.4 ± 0.49	15.3 ± 0.37	30.7 ± 0.55	1.01± 0.03
Structured PC–1.5%	13.6 ± 0.39	13.9 ± 0.61	27.9 ± 0.63	0.98 ±0.03

^g^ Reaction conditions: enzyme loading, 15% (*w*/*w*); substrate mass ratio (PC/ethyl esters), 1:6; temperature, 55 °C; reaction time, 24 h; ^h^ The value (0.5%) means the mass percentage of the water dosage to the total weight of reaction substrates.

### 2.3. Analyzing the Composition of Reaction Product

Under the above optimal conditions, the reaction was performed and the composition of the reaction mixture was analyzed. The results are listed in Table 4. In the original PC, only small amounts of 1-LPC and 2-LPC exist but 97.7% of pure PC (Table 4). It was found that higher content of 1-LPC and 2-LPC was produced after 24 h of reaction, whereas the PC content decreased to be 16.5%, and GPC emerged and accounted for 25.8% of total contents of reaction mixture. As shown in Table 5, the DHA and EPA were successfully incorporated into PC and LPC. The content of DHA and EPA in the structured PC and LPC was 30.8% and 30.3%, respectively.

**Table 4:** Composition of phospholipids of the original PC and structured PC.

Phospholipids	Content (%)	
	Original PC	Structured PC ^i^
PC	97.7 ± 0.45	16.5 ± 0.65
1-LPC	0.4 ± 0.12	26.3 ± 0.58
2-LPC	1.9 ± 0.22	31.4 ± 0.89
GPC	–	25.8 ± 0.72

^i^ Reaction conditions: enzyme loading, 15% (*w*/*w*); substrate mass ratio (PC/ethyl esters), 1:6; temperature, 55 °C; water dosage, 1.25% (*w*/*w*); reaction time, 24 h; “–” means not detected.

### 2.4. Evaluation of Enzyme Reusability

**Figure 7:**
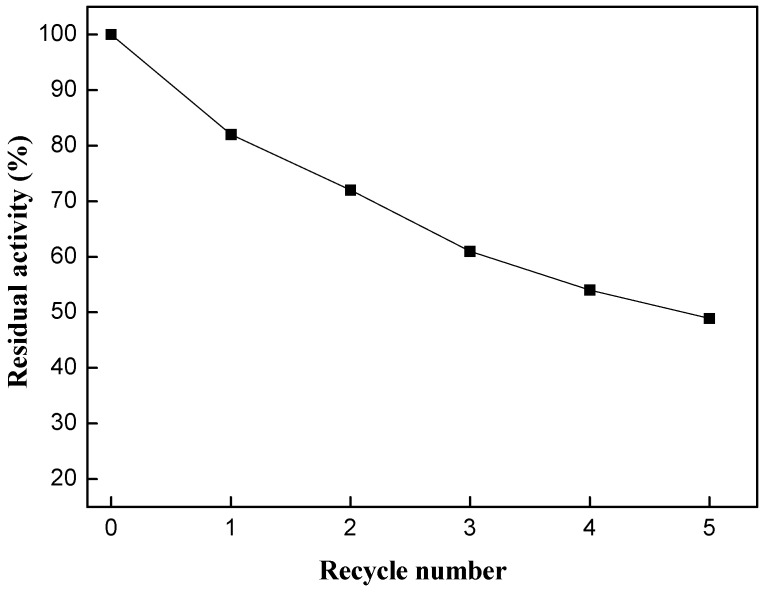
Evaluation of immobilized PLA_1_ reusability. Reaction conditions: enzyme loading, 15% (*w*/*w*); substrate mass ratio (PC/ethyl esters), 1:6; temperature, 55 °C; water dosage, 1.25% (*w*/*w*); reaction time, 24 h.

The reusability of the immobilized enzyme was evaluated under the optimal reaction conditions. The activity of the enzyme was calculated on the basis of the incorporation achieved in 24 h reaction for each circle. The activity of immobilized PLA_1_ decreased to 48.9% after the fifth cycle (after it had been used to be carried out a total of six reactions trials) (Figure 7). It indicated that immobilized PLA_1_ had a good operational stability in the transesterification of PC and ethyl ester-DHA/EPA. Additionally, the immobilization method used in the present work was very simple and cost-effective, so the immobilized PLA_1_ has great potential in the production of structured phospholipids.

**Table 5:** Fatty acid composition (%) of substrate and structured PC.

Substrate				Structured PC ^j^	
Fatty Acid	DHA/EPA-Rich Ethyl Esters	PC ^k^	LPC ^k^	Total PC ^l^	Total LPC ^m^
C14:0	0.2 ± 0.04	1.1 ± 0.05	1.0 ± 0.04	0.5 ± 0.01	0.4 ± 0.03
C16:0	1.1 ± 0.03	13.8 ± 0.13	13.3 ± 0.15	7.1 ± 0.25	8.0 ± 0.22
C16:1	0.2 ± 0.02	0.3 ± 0.01	0.2 ± 0.04	0.3 ± 0.04	0.2 ± 0.03
C18:0	0.6 ± 0.06	3.6 ± 0.08	3.7 ± 0.10	2.1 ± 0.14	2.4 ± 0.08
C18:1	2.2 ± 0.08	10.6 ± 0.18	11.1 ± 0.25	8.4 ± 0.23	8.6 ± 0.12
C18:2	0.6 ± 0.03	64.8 ± 0.32	65.2 ± 0.27	43.1 ± 0.35	42.9 ± 0.31
C18:3	1.4 ± 0.08	5.8 ± 0.07	5.5 ± 0.11	3.8 ± 0.16	3.6 ± 0.08
C22:5	6.4 ± 0.12	–	–	2.5 ± 0.18	2.3 ± 0.14
EPA	38.1 ± 0.24	–	–	15.6 ± 0.25	15.2 ± 0.22
DHA	45.5 ± 0.31	–	–	15.2 ± 0.22	15.1 ± 0.19
EPA + DHA	83.6 ± 0.32	–	–	30.8 ± 0.19	30.3 ± 0.23
Others	3.7 ± 0.11	–	–	1.4 ± 0.10	1.3 ± 0.09

The original PC was composed of 97.7% PC and 2.3% LPC; ^j^ Reaction conditions: enzyme loading, 15% (*w*/*w*); substrate mass ratio (PC/ethyl esters), 1:6; temperature, 55 °C; water dosage, 1.25% (*w*/*w*); reaction time, 24 h; ^k^ PC and ^k^ LPC: the original unmodified PC and LPC; ^l^ Total PC: unreacted and modified PC; ^m^ Total LPC: unreacted LPC that was present in the original reaction mixture plus the LPC formed by hydrolysis and esterification; “–” means not detected.

## 3. Experimental Section

### 3.1. Materials

Granulated soy phosphatidylcholine (PC, purity > 95%, used as reaction substrate) and 1-palmitoyl-*sn*-glycero-3-lysophosphatidylcholine (2-LPC, purity > 99%) were obtained from Avanti Polar-Lipids, Inc. (Alabasta, AL, USA). *sn*-glycerol-3-phosphatidylcholine (GPC, purity > 99%) was purchased from Sigma Chemicals (St. Louis, MO, USA). Soy lecithin (PC, purity > 40%, used for analysis of enzyme activity) was obtained from Shanghai Poly Biotechnology Limited Source (Shanghai, China). Triphenylphosphate (TPP) was purchased from Aladin Reagent (Shanghai, China). Phospholipase A_1_ (Lecitase^®^ Ultra) was provided by Novozymes A/S (Bagsværd, Denmark). Ethyl esters-DHA/EPA (Ethyl esters enriched with 38.1% of EPA and 45.4% of DHA) was kindly provided by Sinomega Biotech Engineering Co., Ltd. (Zhejiang, China). *n*-Hexane of HPLC and deuterated chloroform were purchased from Kermel Chemical Reagent Co., Ltd. (Tianjin, China). The resin D380 was purchased from Chemical Plant of Nankai University (Tianjin, China). Bovine serum albumin (BSA) was purchased from Shanghai Bio Science & Technology Company (Shanghai,China). Bradford reagent was obtained from Sigma (Wuhan, China). All other chemicals were of chromatographic and analytical grade.

### 3.2. Immobilization of Phospholipase A_1_

The commercially available undiluted enzyme solution (Lecitase^®^ Ultra, *ca.* 1.5% protein) was mixed with an equal volume of 0.2 M phosphate buffer (pH 4.0, 5.0, 6.0, 7.0, 8.0). Then, the enzyme solution (12 mL) was added into a 50 mL conical flask containing 2.0 g of macroporous resin, and the flask was placed in a thermostatic air bath shaker (180 r/min) at 30 °C for 6 h. Then the immobilized enzyme was collected by filtration through a Buchner funnel and then washed with 0.2 M phosphate buffer. The process of filtering and washing was repeated several times until no protein was detected in the eluate. The mother liquor and the resulted washing solutions were collected and tested using the Bradford protein assay, the amount of enzyme which is immobilized could be estimated. The conditions such as ratio of enzyme to resin (1–4 mL/g resin) and adoptive pH were optimized and their corresponding immobilized enzyme activity was determined. The immobilized phospholipase A_1_ on macroporous resin was finally lyophilized for 24 h and stored in the closed vials at 4 °C until use.

Determination of protein load of immobilized phospholipase A_1_ was carried out according to the Bradford assay [15]. Protein load was estimated by mean of a calibration curve obtained using BSA as protein standard.

### 3.3. Phospholipase A_1_-Catalyzed Transesterification of PC to DHA/EPA-Rich Ethyl Esters

PC (1.00 g) was mixed with different amount of DHA/EPA-rich ethyl esters (1:3, 1:4, 1:5, 1:6, 1:7, substrate mass ratio of PC to ethyl esters) in 25 mL conical flasks, and then the immobilized PLA_1_ (5%, 10%, 15%, 20%, 25% *w*/*w*, with respect to total reaction mixture) and water dosage (0.5%, 0.75%, 1.0%, 1.25%, 1.5% *w*/*w*, with respect to total reaction mixture) were subsequently added in the substrates. The mixture was incubated at various temperatures (40, 45, 50, 55, 60 °C) in a shaking air bath at 200 r/min. Individual samples were withdrawn at selected times and analyzed.

### 3.4. Evaluation of Enzyme Reusability

The reusability of the employed immobilized PLA_1_ system was evaluated to ascertain the stability of biocatalysts in the transesterification reaction by recovering and transferring the enzyme to a fresh substrate mixture. The reactions were carried out under the optimal reaction conditions. After a time corresponding to one reaction cycle (24 h), the immobilized PLA_1_ was filtered out and recycled for the next cycle. The remaining activity was measured in terms of the decrease of incorporation for each cycle. The point corresponding to cycle 0 corresponds to the activity of the immobilized PLA_1_ used in the first reaction trial (taken as 100%).

### 3.5. Analyzing Activity of Immobilized PLA_1_

Phospholipase activity was determined according to the method of Yang *et al.* [16]. Substrate solution: 40 g of PL (deoiled soy lecithin, PC > 40%) and 1000 mL of 0.2 M phosphate buffer solution (pH 4.0, 5.0, 6.0, 7.0, 8.0) (prepared using 2% polyvinyl alcohol) were emulsified. One unit of hydrolysis activity (U) is the amount of enzyme which releases 1 μmol of titratable free fatty acids (FFA) per minute under the described conditions.

### 3.6. Analyzing Moisture Content of Immobilized PLA_1_

The moisture content of lyophilized immobilized PLA_1_ was detected by IR-35 moisture analyzer at 105 °C for 10 min.

### 3.7. Analyzing FA Composition by Gas Chromatography (GC)

(1) Preparation of phospholipid precipitation from reaction mixture: after reactions were suspended, PC precipitation was performed according to the method developed by Marsaoui *et al.* [8]. The final precipitated pellet was dried under vacuum (0.01 MPa, 30 min) and stored at −40 °C under a N_2_ atmosphere until use for GC analysis; (2) Separation of LPC and PC from reaction mixture: After reaction, 100 μL of reaction mixture was withdrawn and was diluted in 1 mL of chloroform. And then the mixture were applied to thin layer chromatography (TLC) plates (10 × 20 cm) coated with silica gel G, and was developed in a TLC tank. The developing solvent was chloroform/methanol/acetic acid/water (75:40:8:3, *v*/*v*/*v*). The bands were sprayed with 0.2% 2,7-dichlorofluorescein in methanol and visualized under ultraviolet (UV) light. The PC and LPC band was scraped off for the analysis of fatty acid composition; (3) Analyzing the fatty acid composition of precipitated phospholipids and the separated LPC and PC by GC: Samples such as precipitated phospholipids, PC and LPC band were methylated to FA methyl esters according to the method of ISO 5509:2000(E) [17] and then were analyzed on an Agilent 7890A gas chromatograph (GC) equipped with a capillary column CP-Sil 88 (60 m × 0.25 mm × 0.2 μm; Dikma Technologies, Beijing, China) [18]. In our study, the incorporation of FA (%) was calculated as follows (Equation (1)):

Incorporation of FA= (FA content in PL/Total FA content in PL) × 100%
(1)

### 3.8. Analyzing Structured PC Composition in Reaction Products by ^31^P NMR

Phospholipids precipitation was separated according to the above method, and then its composition was analyzed by ^31^P NMR. All NMR experiments were conducted on a Bruker AV600 spectrometer (Bruker BioSpin, Billerica, MA, USA) operating at 243 MHz for phosphorus-31 nuclei, at 25 °C using temperature stabilization. An amount of 50 mg of the extracted phospholipids precipitation obtained from reaction mixtures was dissolved in 0.6 mL of CDCl_3_/MeOH (2:1 *v*/*v*). The mixture was added directly into the 5 mm NMR tube and used to obtain the NMR spectra. These spectra were recorded by employing the inverse gated decoupling technique to suppress NOE (Nuclear Overhauser Effect). Parameters were as follows: pulse width, 12 μs; acquisition time, 2.3 s; repetition time (relaxation delay + acquisition time), 7.3 s; number of scans, 96. The large sweep width was dictated by the ^31^P chemical shift (δ −17.80) of the internal standard triphenylphosphate (TPP). The data processing was completed with MestReNova software (Mestrelab Research SL, Santiago de Compostela, Spain). Typical chemical shift values obtained (relative to TPP as an internal standard) were as follows: δ −17.8 (TPP), −0.84 (PC), −0.32 (1-LPC), −0.18 (2-LPC), and 0.02 ppm (GPC). The relative integrated intensity (*I*) of each peak was used to calculate the composition of the product mixture.

### 3.9. Statistical Analysis

All experiments were performed in triplicate and results were given as mean ± SD. Significant differences in the means were accomplished by using an ANOVA procedure (*p* < 0.05).

## 4. Conclusions

That enzymatic transesterification of DHA/EPA-rich ethyl esters and PL was shown to be a good system for lipid modification. The system showed good solubility with no need for solvent which is suitable in the food processing for non-toxic contamination. Compared with liquid PLA_1_, immobilized PLA_1_ exhibited better catalytic efficiency and more preference to EPA than DHA in the transesterification. The immobilized PLA_1_ was used to catalyze the transesterification of PC and ethyl ester-DHA/EPA, and the maximal incorporation was 30.7% under the optimized conditions: enzyme loading at a concentration of 15% (*w*/*w*, with respect to total reaction mixture), PC/ethyl esters mass ratio of 1:6, temperature of 55 °C, and water dosage of 1.25% (*w*/*w*, with respect to total reaction mixture). We confirmed that the immobilized enzyme had a good reusability.

The reaction product was analyzed by ^31^P NMR, and it was found that was a mixture of PC (16.5%), 1-LPC (26.3%), 2-LPC (31.4%) and GPC (25.8%). About 74% of the total yield of structured PC (DHA/EPA-rich PC and DHA/EPA-rich LPC) reached in our present study. Although hydrolysis reaction was significant in both transesterification (present study) and acidolysis (reported by Kim *et al.* [9]) leading to a low yield of DHA/EPA-rich PC, DHA/EPA-rich LPC has been also reported to have their special function in food, cosmetics and pharmaceutical industries. Thus, the immobilized PLA_1_ has potential for catalyzing the transesterification to produce various structured lipids including DHA/EPA-rich PC and DHA/EPA-rich LPC by controlling the reaction conditions according to the specific application of interest in different industries.
